# Need for pacing in patients who qualify for an implantable cardioverter‐defibrillator: Clinical implications for the subcutaneous ICD

**DOI:** 10.1111/anec.12744

**Published:** 2020-01-29

**Authors:** Valentina Kutyifa, Spencer Z. Rosero, Scott McNitt, Bronislava Polonsky, Mary W. Brown, Wojciech Zareba, Ilan Goldenberg

**Affiliations:** ^1^ Heart Research Follow‐Up Program University of Rochester Medical Center Rochester NY USA

**Keywords:** MADIT‐II, pacemaker, pacing, PR interval, subcutaneous implantable cardioverter‐defibrillator

## Abstract

**Background:**

Implantation of the subcutaneous implantable cardioverter‐defibrillator (S‐ICD) is spreading and has been shown to be safe and effective; however, it does not provide brady‐pacing. Currently, data on the need for brady‐pacing and cardiac resynchronization therapy (CRT) implantation in patients with ICD indication are limited.

**Methods:**

The Multicenter Automatic Defibrillator Implantation Trial (MADIT)‐II enrolled post‐MI patients with reduced ejection fraction (EF ≤ 35%), randomized to either an implantable cardioverter‐defibrillator (ICD) or conventional medical therapy. Kaplan–Meier analyses and multivariate Cox models were performed to assess the incidence and predictors of pacemaker (PM), or CRT implantation in the conventional arm of MADIT‐II, after excluding 32 patients (6.5%) with a previously implanted PM.

**Results:**

During the median follow‐up of 20 months, 24 of 458 patients (5.2%) were implanted with a PM or a CRT (19 PM, 5 CRT). Symptomatic sinus bradycardia was the primary indication for PM implantation (*n* = 9, 37%), followed by AV block (*n* = 5, 21%), tachy‐brady syndrome (*n* = 4, 17%), and carotid sinus hypersensitivity (*n* = 1, 4%). Baseline PR interval >200 ms (HR = 3.07, 95% CI: 1.24–7.57, *p* = .02), and CABG before enrollment (HR = 6.88, 95% CI: 1.58–29.84, *p* = .01) predicted subsequent PM/CRT implantation. Patients with PM/CRT implantation had a significantly higher risk for heart failure (HR = 2.67, 95% CI = 1.38–5.14, *p* = .003), but no increased mortality risk (HR = 1.06, 95% CI = 0.46–2.46, *p* = .89).

**Conclusion:**

The short‐term need for ventricular pacing or CRT implantation in patients with MADIT‐II ICD indication was low, especially in those with a normal baseline PR interval, and such patients are appropriate candidates for the subcutaneous ICD.

## 1 INTRODUCTION

The Multicenter Automatic Defibrillator Implantation Trial II (MADIT‐II) was a randomized clinical trial investigating the role of the implantable cardioverter‐defibrillator (ICD) in postmyocardial infarction (MI) patients with severely reduced left ventricular ejection fraction (LVEF ≤ 35%). The primary results of MADIT‐II showed ICD implantation to reduce all‐cause mortality when compared to conventional medical treatment (Shah et al., 2010).

Today, thousands of patients are implanted with ICD based on the MADIT‐II indication. More recently, the introduction of the subcutaneous implantable cardioverter‐defibrillator (S‐ICD) revolutionized the field by introducing a defibrillator system without an implanted lead in the heart (Sanghera, Sanders, Husby, & Bentsen, 2014). Implantation of subcutaneous implantable cardioverter‐defibrillator (S‐ICD) is spreading worldwide and has been shown to be safe and effective to terminate life‐threatening ventricular tachyarrhythmias (Burke et al., 2015; Gold et al., 2017). Recent propensity‐matched studies also suggested that the S‐ICD has similar complication rates as the transvenous ICD, but less lead‐related complications, and a much lower cost of complications (Brouwer et al. (2016); Brouwer et al., 2018). However, the S‐ICD does not currently provide brady‐pacing. Currently, data on the need for brady‐pacing and the need for cardiac resynchronization therapy (CRT) in patients with MADIT‐II ICD indication are limited.

Therefore, the aim of the current MADIT‐II substudy was (a) to evaluate the need for brady‐pacing and the need for cardiac resynchronization therapy in patients in the conventional medical treatment arm in MADIT‐II, (b) to identify baseline predictors of brady‐pacing and indication for subsequent cardiac resynchronization therapy, and (c) to evaluate the risk of subsequent heart failure and mortality in patients undergoing PM/CRT implantation, enrolled in the conventional medical treatment arm in MADIT‐II.

## 2 METHODS

### 2.1 Study population

The design and primary results of the MADIT‐II trial were previously published (Moss et al., 2002). Briefly, the MADIT‐II trial was designed to determine the effectiveness of the implantable cardioverter‐defibrillators to reduce all‐cause mortality in patients with reduced left ventricular function following a myocardial infarction. Subjects over the age of 21 years who had a documented myocardial infarction 1 month or more before study enrollment and a low left ventricular ejection fraction of less than ≤35% were enrolled in MADIT‐II. Patients were randomly assigned in a 3:2 ratio to either ICD or conventional medical therapy. The primary end point of MADIT‐II was death from any cause. Clinical data and interrogation data from the ICD devices were sent to the study Coordination and Data Center (CDC) at the University of Rochester, Heart Research Follow‐Up Program, Rochester, New York.

The current study included 458 patients (37%) randomized to the conventional medical treatment arm of MADIT‐II, with no implanted ICDs. We excluded 32 patients (6.5%) from the analyses who had previously implanted pacemaker devices before enrollment in MADIT‐II.

### 2.2 End points and definitions

Subjects enrolled in MADIT‐II were followed for an average of 20 months. A pacemaker or CRT implantation for any reason was the primary end point of the current analysis. Secondary end point was subsequent heart failure events and all‐cause mortality following pacemaker or CRT implantation. Adverse event reports were individually reviewed for evidence of pacemaker or CRT implantation during the course of the trial by the primary author, Dr. Valentina Kutyifa.

### 2.3 Statistical analysis

Continuous variables are expressed as mean ± *SD*. Categorical data are summarized as frequencies and percentages. Baseline clinical characteristics were compared between patients with or without an implanted PM/CRT during the follow‐up, using Wilcoxon rank sum test for continuous variables and chi‐square test for dichotomous variables.

Kaplan–Meier survival analyses were performed to assess the cumulative probability of PM/CRT implantation during the course of the trial in the total patient population, as well as by baseline PR interval, and by prior CABG prior to enrollment with comparisons between groups using the log‐rank test. Multivariate Cox proportional hazards regression analysis was used to evaluate the predictive factors of PM or CRT implantation during follow‐up. Best subset regression modeling was utilized to select the variables. Candidate covariates were considered as shown in Table 1. A second set of multivariate Cox proportional hazards regression model was performed to assess the effects of time‐dependent PM/CRT implantation on subsequent heart failure events and all‐cause mortality, adjusted for relevant baseline covariates.

**Table 1:** Baseline Clinical Characteristics of Patients with and without Subsequent PM/CRT

	PM/CRT	No PM/CRT	*p*‐Value
# of patients	24	434	
Age At Randomization (years)	68 ± 9	64 ± 10	.155
Female	1 (4)	68 (16)	.152
White Race	23 (96)	373 (86)	.228
Diabetes	11 (46)	157 (36)	.344
Hypertension Requiring Treatment	12 (50)	233 (54)	.716
NYHA Class II‐IV Before Enrollment	18 (75)	259 (61)	.160
Non‐CABG Revascularization	10 (42)	183 (43)	.931
Coronary Bypass Surgery	21 (88)	230 (53)	**<.001**
Atrial Fibrillation	5 (21)	36 (8)	.056
Left Bundle Branch Block	7 (29)	71 (17)	.160
Right Bundle Branch Block	3 (13)	26 (6)	.197
Beta‐blockers (baseline)	16 (67)	263 (61)	.553
Digitalis (baseline)	17 (71)	239 (55)	.130
ACE‐Inhibitor (baseline)	18 (75)	340 (78)	.700
Angiotensin Receptor Blockers (baseline)	2 (8)	53 (12)	.755
Amiodarone (baseline)	2 (8)	28 (6)	.665
Diuretic (baseline)	20 (83)	335 (77)	.483
EF < 25%	14 (58)	199 (46)	.233
QRS (ms)	140 ± 32	116 ± 29	**<.001**
PR Interval (ms)	221 ± 44	193 ± 39	**.003**
Blood Urea Nitrogen (mM)	25 ± 10	23 ± 12	.292
Creatinine (mg/dl)	1.3 ± 0.2	1.3 ± 0.6	.143
Occurrence of New/Worsening CHF Req. Hosp.	12 (50)	66 (15)	**<.001**
New/Worsening CHF Req. Hosp. or Death	13 (54)	122 (29)	**.008**

A *p*‐value of < .05 was statistically significant and bolded.

All statistical tests were two‐sided, and a *p*‐value of < .05 was considered statistically significant. Interaction *p*‐values were computed and reported. Analyses were carried out with SAS software (version 9.3, SAS institute).

## 3 RESULTS

**Figure 1:**
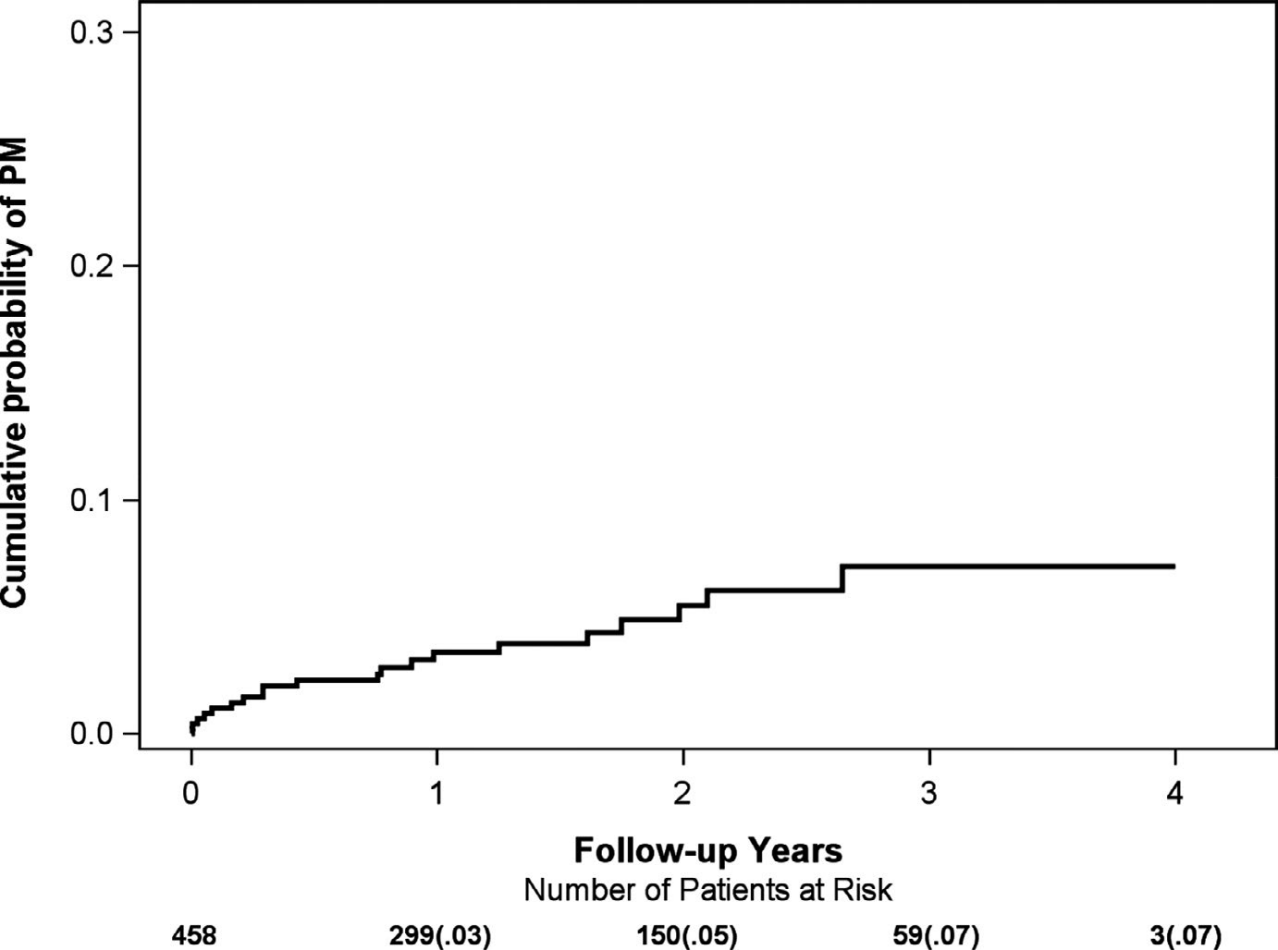
Cumulative Probability of PM/CRT implantation in MADIT‐II

During the median follow‐up of 20 months in MADIT‐II, there were a total of 24 out of 458 patients (5.2%) implanted with a PM or a CRT device. Five of these patients (21%) received a CRT device due to heart failure symptoms. The cumulative probability of PM/CRT implantation was 3% at 1 year, 5% at 2 years, and 7% at 3 years of follow‐up (Figure 1).

### 3.1 Indication for Brady‐pacing in MADIT‐II

Symptomatic sinus bradycardia was the primary indication for PM implantation (*n* = 9, 37%), followed by AV block (*n* = 5, 21%), tachy‐brady syndrome (*n* = 4, 17%), and carotid sinus hypersensitivity (*n* = 1, 4%). Cardiac resynchronization therapy implantation was necessitated by an acute heart failure event in all five patients.

### 3.2 Baseline clinical characteristics

Clinical characteristics of patients with or without an implanted PM/CRT during the follow‐up are shown in Table 1. Patients with an implanted PM/CRT were older, but the difference did not reach statistical significance (68 vs. 64 years, *p* = .155). Prior history of coronary artery bypass graft surgery (CABG) was significantly more frequent in PM/CRT recipients (88% vs. 53%). Patients with the need for brady‐pacing or CRT had a significantly longer QRS duration (140 vs. 116 ms) and a longer PR interval at baseline (221 vs. 193 ms). Interestingly, a prior history of atrial fibrillation was also more frequent in the PM/CRT recipients group (21% vs. 8%).

### 3.3 Predictors of PM/CRT Implantation

**Figure 2:**
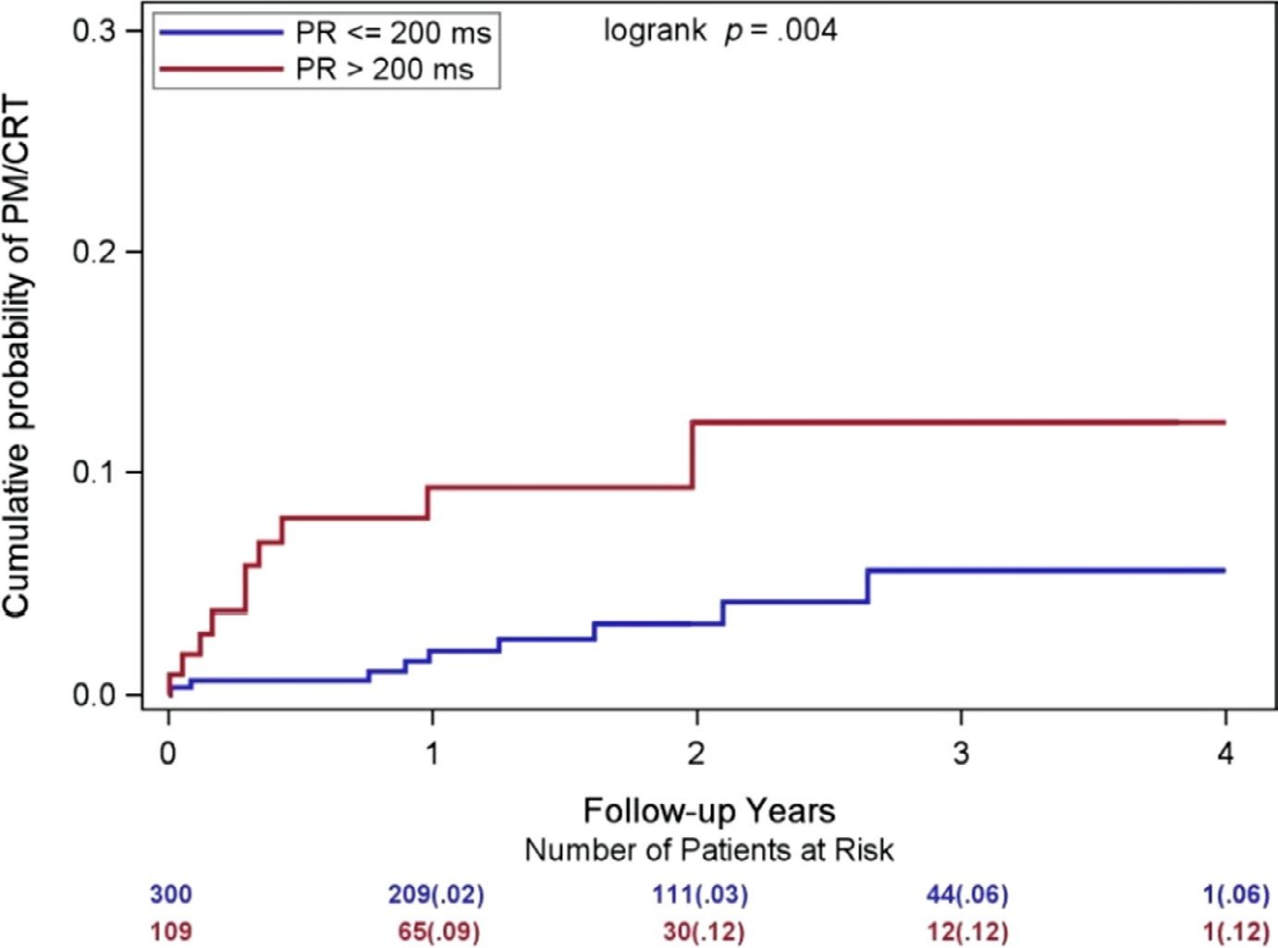
Cumulative Probability of PM/CRT implantation by baseline PR interval

When assessing baseline predictors for subsequent PM/CRT implantation in MADIT‐II, we found that a prolonged baseline PR interval >200 ms was linked to a threefold increase in the risk of PM/CRT implantation (*p* = .02; Table 2). The cumulative probability of PM/CRT implantation in patients with a normal baseline PR interval ≤200 ms at baseline was 2% at 1 year, and 6% at 3 years, as compared to the cumulative probability of 9% at 1 year, and 12% at 3 years in patients with PR interval >200 ms (Figure 2). Interestingly, the majority of the PM/CRT implantations occurred in this group in the first year of follow‐up (9% 1‐year cumulative probability).

**Table 2:** Predictors of pacemaker/ CRT implantation

Pacemaker/CRT implantation during the follow‐up			
Parameter	Hazard ratio	95% CI	*p*‐Value
Baseline PR > 200 ms	3.07	1.24 – 7.57	.02
CABG before enrollment	6.88	1.58 – 29.84	.01

Abbreviation: CABG, coronary artery bypass graft surgery.

**Figure 3:**
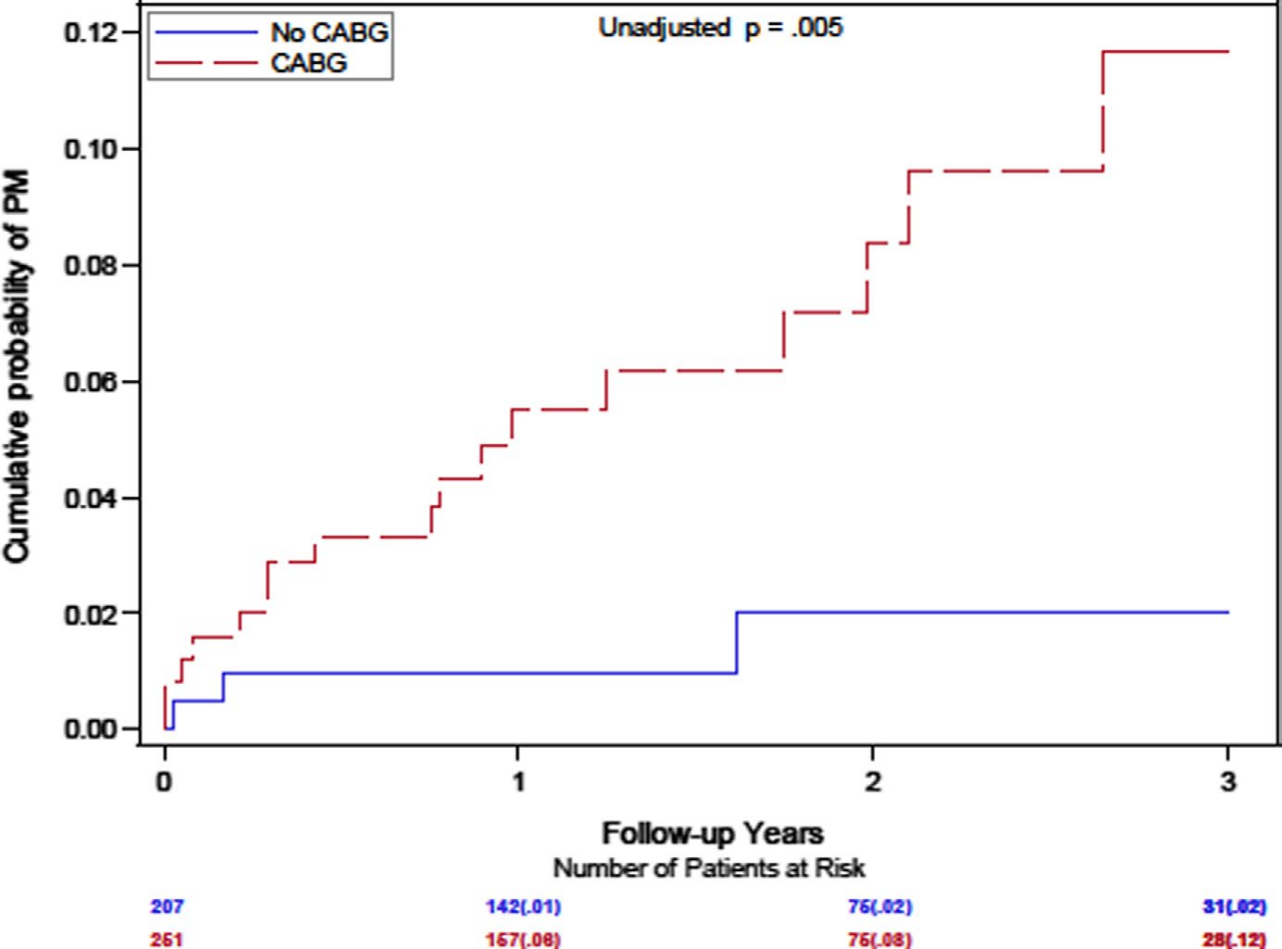
Cumulative Probability of PM/CRT implantation by History of CABG

In addition, a prior history of CABG was associated with a significant, almost sevenfold increase in the likelihood of PM/CRT implantation during follow‐up (*p* = .01; Table 2). The cumulative probability of PM/CRT implantation at 1 year was 8% versus 1%, and 12% versus 2% at 3 years with or without a prior CABG (Figure 3).

### 3.4 Risk of heart failure and all‐cause mortality following PM/CRT implantation

Following a PM or CRT implantation, there was a significant, 2.67‐fold increased risk of subsequent HF events observed, following adjustments for relevant clinical covariates. However, the risk of all‐cause mortality was not significantly higher following the PM/CRT implantation (HR = 1.06, *p* = 089, Table 3).

**Table 3:** Clinical outcome after PM/CRT implantation

Clinical outcome after PM/CRT implantation			
	Hazard Ratio	95% CI	*p*‐Value
Heart failure	2.67	1.38 – 5.14	.003
All‐cause mortality	1.06	0.46 – 2.46	.89

Note

Models were adjusted for blood urea nitrogen, and prior hospitalization.

### 3.5 Sensitivity analyses

We have also performed sensitivity analysis predicting PM implantation while excluding patients with CRT indication during the follow‐up, and we had consistent findings (Prior CABG: HR = 11.57, *p* = .018, and PR > 200 ms: HR = 2.40, *p* = .09).

## 4 DISCUSSION

In this substudy of MADIT‐II, we show that the need for pacing or CRT implantation was ~2% per year, and it was predicted by a prolonged PR interval >200 ms at baseline, and by the history of prior CABG. Patients with a PM/CRT implantation in MADIT‐II had a significantly higher risk of subsequent heart failure events, but the risk of all‐cause mortality was not increased. Altogether our findings suggest a low rate for bradycardia pacing/CRT implantation in primary prevention ICD patients.

There have been a few previous studies that evaluated the need for bradycardia pacing or CRT implantation in ICD patients. Poole et al. showed that in the SCD‐HeFT study, the 5‐year rate of pacing need was 10%. This rate is consistent with our observation of ~2% annual rate for the need for pacing in a primary prevention ICD population. Another recent study by Melles et al. similarly suggest a low overall rate of the need for atrial or ventricular pacing, and of developing a CRT indication, with 1.8 (95% CI 1.2 to 2.6) events per 100 patient‐years for CRT, and 0.3 events (95% CI 0.1 to 0.8) per 100 patient‐years for pacing dependency (Melles et al., 2018). This is especially relevant at the time of emerging new technologies, such as the subcutaneous implantable cardioverter‐defibrillator (S‐ICD) that does not provide pacing and, therefore, should not be the choice of therapy in such patients.

Identifying patients at high risk for pacing following a primary prevention ICD implantation is crucial to address this knowledge gap. In our study, we report a number of useful pre‐implant risk factors identifying patients at risk for pacing/CRT indication development. Patients developing pacing need/CRT indication were older, they more often had prior CABG, a history of atrial fibrillation, and they had longer PR intervals and QRS duration. Independent risk factors emerged to be a prolonged PR interval and a history of CABG. A prolonged AV interval has previously shown to be associated with an increased risk of heart failure events, atrial fibrillation, and all‐cause mortality (Barold, Ilercil, Leonelli, & Herweg, 2006; Kwok & Rashid M et al., 2016; Magnani et al., 2013). In addition, a prolonged AV interval has been frequently reported in heart failure patients (~10%), and it was linked to an unfavorable outcome in cardiac resynchronization therapy candidates, unless they had non‐LBBB (Cleland et al., 2008; Kutyifa et al., 2014; Olshansky et al., 2012; Salden, Kutyifa, Stockburger, Prinzen, & Vernooy, 2018). Nevertheless, a study by Uhm et al. suggested that PR interval is associated not only with atrial fibrillation and left ventricular dysfunction, but also with an increased risk of advanced atrioventricular block (Uhm et al., 2014), explaining why patients in our cohort more frequently developed a need for pacing in the prolonged AV‐interval subgroup. Importantly, in our study, a prolonged PR interval predicted not only the need for pacing but also the development of heart failure events and a need for CRT, suggesting that it is a marker for multiple outcomes.

We need to acknowledge, however, that at the time of the MADIT‐II trial, beta‐blockers were less frequently used as they are today. In MADIT‐II, beta‐blockers were administered in 61%–67% while in recent ICD trials, this rate is 90%–95% (Gasparini et al., 2013; Moss et al., 2012). Therefore, the need for pacing in MADIT‐II might be underestimated. More contemporary databases of ICD patients could be potentially helpful to ascertain the need for pacing with optimal medical treatment for HF.

There have been a number of randomized clinical trials on the effectiveness of cardiac resynchronization therapy completed since MADIT‐II (Bristow et al., 2004; Cleland et al., 2005; Linde, Abraham, Gold, & Daubert, 2010; Moss et al., 2009; Tang et al., 2010), establishing CRT as a guideline‐indicated therapy for both advanced, and mild HF patients (Epstein et al., 2013) with a wide QRS. The impact of these indications could not be investigated in this substudy, and therefore, we are likely underestimating the need for CRT in this population.

Our findings are in alignment with another recent study that looked at the pacing percentage in primary prevention ICD patients as a surrogate for the need for pacing in this cohort. Kalantarian et al. (2018) concluded from a large cohort of 1635 VVI ICD patients derived from the NCDR ICD database (programmed VVI mode 40 bpm at implant) that >5% pacing need was present in 6.6% over 2 years (~3% annual rate) in primary prevention ICD patients. Their study identified older age (age >80 years), and the presence of atrial fibrillation prior or at the implantation as independent risk factor for the need for subsequent pacing.

Altogether, our study with previous observations suggests using simple, clinical risk factors such as age, prior CABG, atrial fibrillation, and prolonged PR interval to identify patients at higher risk of pacing who might not be eligible for an S‐ICD. Nevertheless, a large proportion of the currently implanted primary prevention ICDs would be eligible for an S‐ICD with a favorable risk profile. However, further studies are needed to prospectively assess the performance of these suggested risk factors.

Interestingly, we also showed that a PM/CRT implantation predicted subsequent HF events probably due to the underlying left ventricular dysfunction or due to frequent RV pacing, as previous studies proposed (Kutalek, Sharma, & McWilliams, 2008; Sweeney et al., 2003). However, a PM/CRT implantation was not an indicator of an increased subsequent mortality risk. This suggests that low‐risk patients with low likelihood of developing a pacing need or need for CRT, an S‐ICD strategy combined with regular assessment for the need for subsequent pacing/CRT might be sufficient, and it would not adversely affect mortality. Nevertheless, this needs to be further tested in prospective studies.

We have to acknowledge a number of limitations of the current study. In MADIT‐II, PM/CRT implantations were reported as adverse events during the follow‐up, and therefore, limited information was available on the clinical circumstances prompting these device implantations. At the time of conducting MADIT‐II, CRT was not yet readily available, thereby potentially limiting the number of patients considered for CRT. In MADIT‐II, use of beta‐blockers was 61%–67%, much lower than that of what we would normally see in a similar patient population. Underreporting of PM/CRT implantation events might be another limiting factor. In addition, follow‐up time in MADIT‐II was an average of 20 months, and therefore, the long‐term need for pacing cannot be fully ascertained. However, we still have a very large, unique cohort of patients with MADIT‐II ICD indication who were not implanted with an ICD.

## 5 CONCLUSIONS

The need for ventricular pacing or CRT implantation in MADIT‐II patients was low, ~2% per year, especially in those with normal PR interval and no prior CABG, and in such patients, an S‐ICD can be considered for primary prevention ICD indication. However, a large proportion of these patients are currently indicated for CRT implantation, and the need for pacing might be underestimated in MADIT‐II due to the low proportion of beta‐blocker use.

## CONFLICT OF INTEREST

Dr. Kutyifa ‐ use the text in the manuscript. Drs. Zareba and Goldenberg received research grants from Boston Scientific. The protocol was approved by the institutional review boards of the participating hospitals. All patients provided an informed consent.

## AUTHORS CONTRIBUTIONS

VK ‐ Conceptualization; Investigation; Project administration; Supervision; Visualization; Roles/Writing ‐ original draft; Writing ‐ review & editing. SR ‐ Writing ‐ review & editing. SM ‐ Data curation; Formal analysis; Methodology; Writing ‐ review & editing. BP ‐ Data curation; Formal analysis; Methodology. MB ‐ Data curation; Supervision; Methodology. WZ ‐ Supervision, Writing ‐ review & editing. IG ‐ Supervision, Writing ‐ review & editing.

## DISCLOSURES

Dr. Kutyifa received research grants from Boston Scientific, ZOLL Inc., Biotronik, and Spire, consultant fees from Biotronik, ZOLL Inc., and travel support from ZOLL Inc.
